# Next Generation Sequencing Identifies the HLA-DQA1*03:03 Allele in the Type 1 Diabetes Risk-Associated HLA-DQ8 Serotype

**DOI:** 10.3390/genes12121879

**Published:** 2021-11-25

**Authors:** Jürgen Enczmann, Vera Balz, Maximilian Hoffmann, Sebastian Kummer, Christina Reinauer, Carsten Döing, Katharina Förtsch, Alena Welters, Ertan Mayatepek, Thomas Meissner, Marc Jacobsen, Julia Seyfarth

**Affiliations:** 1 Institute for Transplantation Diagnostics and Cell Therapeutics, Medical Faculty, University Hospital, Heinrich-Heine-University Duesseldorf, 40225 Duesseldorf, Germany; Juergen.Enczmann@med.uni-duesseldorf.de (J.E.); Balz@med.uni-duesseldorf.de (V.B.); 2 Department of General Pediatrics, Neonatology and Pediatric Cardiology, Medical Faculty, University Hospital, Heinrich-Heine-University Duesseldorf, 40225 Duesseldorf, Germany; Maximilian.Hoffmann@uni-duesseldorf.de (M.H.); sebastian.kummer@med.uni-duesseldorf.de (S.K.); christina.reinauer@med.uni-duesseldorf.de (C.R.); Carsten.Doeing@med.uni-duesseldorf.de (C.D.); Katharina.Foertsch@med.uni-duesseldorf.de (K.F.); Alena.Welters@med.uni-duesseldorf.de (A.W.); mayatepek@med.uni-duesseldorf.de (E.M.); thomas.meissner@med.uni-duesseldorf.de (T.M.); marc.jacobsen@med.uni-duesseldorf.de (M.J.)

**Keywords:** HLA, type 1 diabetes, HLA-DQ, next generation sequencing, type 1 diabetes risk

## Abstract

The highest genetic type 1 diabetes risk is conferred by HLA class II haplotypes defined by alleles at the *HLA-DR* and -*DQ* loci. The combination of HLA-DQA1*03:01 and DQB1*03:02 alleles (summarized as ‘HLA-DQ8′) is reported to be among the two most prevalent HLA class II haplotypes in Caucasian type 1 diabetes patients. This classification is based on conventional genotyping of exon 2 of the DQ gene locus and excludes exon 3. In this study, HLA genotyping on the type 1 diabetes susceptibility loci *HLA-DRB1*, *DQA1* and *DQB1* was performed using a high-resolution next generation sequencing method. In addition to the routinely examined exon 2, exon 3 was also sequenced. Samples from 229 children with type 1 diabetes were included and compared to a cohort of 9,786 controls. In addition to previously described HLA-DQ haplotypes in type 1 diabetes patients, we found that as well as HLA-DQA1*03:01, HLA-DQA1*03:03 also contributed to HLA-DQ8. HLA-DQA1*03:03 differs from HLA-DQA1*03:01 by one nucleotide substitution in exon 3 at position 160, leading to a single amino acid replacement. DRB1*04:05 was exclusively associated with DQA1*03:03 whereas the DRB1*04:01 haplotype comprised either DQA1*03:01 or DQA1*03:03. Significantly increased type 1 diabetes risk was confirmed for all these haplotypes with only minor differences between DQA1*03:01 and DQA1*03:03 alleles. This study identified the HLA-DQA1*03:03 allele as an addition to the already known type 1 diabetes risk haplotypes, and can contribute to more precise HLA genotyping approaches.

## 1. Introduction

HLA molecules have been associated with the etiology of several autoimmune diseases including type 1 diabetes [1]. Mechanistically, it is assumed that HLA genotype-associated changes in the shape and charges of the peptide-binding groove determine the preferred repertoire of peptides that can bind and are subsequently presented to T cells [2]. 

In type 1 diabetes, the HLA class II association is the strongest of all genetic associations, leading to either reduced or increased disease susceptibility [1]. Combinations of the *HLA-DRB1, -DQA1*, and *-DQB1* alleles strongly affect the risk of type 1 diabetes, and the highest risk genotype has an odds ratio of >16 [3,4]. The DR4/DQ8 haplotype is (together with the DR3/DQ2 haplotype) part of the prevailing HLA genotype in type 1 diabetes. At least one of these haplotypes is found in almost 90% of children diagnosed with type 1 diabetes [5]. DR4/DQ8 is the common abbreviation for the haplotype combining the alleles DRB1*04-DQA1*03:01-DQB1*03:02. The simplifying classification DR4/DQ8, made according to HLA serotypes, dates to a time when only major antigenic HLA epitopes could be distinguished using serological methods. Nowadays, HLA-typing is carried out through DNA-based molecular techniques, ranging from probe/primer-based techniques to next generation sequencing-based methods [1]. This enables accurate classification of HLA subtypes, which is essential for their evaluation as protective or susceptible haplotypes of varying degrees, as demonstrated for DR4 [6]. However, even with comprehensive HLA genotyping, only exon 2 of the HLA class II molecules is routinely sequenced; exon 2 forms the peptide-binding region of the corresponding HLA molecules and contains most of the polymorphic sites in HLA genes [7,8]. Exon 3 also encodes the extracellular domain of the molecule, but this part does not come into contact with the peptide and is usually not investigated [1]. In this study, we performed HLA typing of type 1 diabetes risk-associated HLA class II molecules in a cohort of 229 pediatric individuals with type 1 diabetes by next generation sequencing of both exons 2 and 3. Here we report differences compared to the previously described *DQA1* alleles as part of the type 1 diabetes risk-associated DR4/DQ8 haplotype.

## 2. Materials and Methods

### 2.1. Donor Characteristics

The study cohort of children and adolescents with type 1 diabetes (*n* = 229, median age 12.2 years (range 1.8–17.9 years), 47.6% females) was recruited at University Children’s Hospital in Duesseldorf, Germany. Written informed consent was received from all study participants (older than 14 years) and their legal guardians.

To estimate the DQA1*03:01 and DQA1*03:03 distribution in the DR4/DQ8 haplotypes, DQB1*03:02-positive samples from healthy donors with different DRB1*04 alleles were selected. The number of samples with common DRB1*04 alleles were limited to reduce the number of samples to be analyzed. The samples mainly came from the Bone Marrow Donor Registry Düsseldorf, Germany. To analyze as many different DRB1*04 alleles as possible, samples from other sources (e.g., external quality controls) were included. Overall, 279 samples with 19 different DRB1*04-alleles were included in the analysis. DRB1–DQA1–DQB1 haplotypes were determined using the assumption that the *DRB1* allele is linked to DQB1*03:02 and DQA1*03.

To compare the frequency of DQA1*03:03-containing type 1 diabetes risk haplotypes between healthy controls and individuals with type 1 diabetes, we used a control population comprising of 9786 samples of randomly selected stem cell donors of the Bone Marrow Donor Registry Düsseldorf. These donors had been DRB1–DQB1 HLA-typed using next generation sequencing during the year 2015. DRB1–DQB1 haplotype frequencies were calculated using the Arlequin software package (http://cmpg.unibe.ch/software/arlequin35/, version 3.5.2.2, accessed on 15 August 2015). The distribution of DQA1*03:01/DQA1*03:03 alleles in the DR4/DQ8 haplotypes of this control population was then estimated using the DQA1*03:03 proportions of the sequenced DR4/DQ8 positive donors.

All methods were performed in accordance with relevant guidelines and regulations.

### 2.2. HLA Typing

Genomic DNA was extracted from heparin blood samples using the DNAQiamp 96 DNA Blood kit (Qiagen, Hilden, Germany) according to the manufacturer’s instructions.

For genotyping of *HLA-DRB1, -DQA1* and *-DQB1*, we chose an amplicon-based approach using Illumina next generation sequencing technology. Primers were designed to target exons 2 and 3 for *HLA-DRB1, -DQA1,* and *–DQB1*. Amplificates are comprised of each entire exon and their additional flanking intron sequences. All primers were screened for additional SNPs using the SNPCheck software (https://genetools.org/SNPCheck/snpcheck.htm, accessed on 11 May 2021). Additional SNPs may impair primer binding and, consequently, result in allele drop-out and erroneous genotyping. Primers were purchased from Biolegio (Nijmegen, The Netherlands). Each primer pair was checked for specificity using Sanger sequencing.

The entire set of fragments was amplified in three multiplex PCR reactions. After a clean-up step using paramagnetic beads, sample-specific barcodes and Illumina compatible adapter sequences were added in a second-round PCR. The samples were pooled, underwent a second purification step, and were quantified using the QuantiFluor dsDNA system (Promega, Walldorf, Germany). Seven pM of the NGS library were applied to the MiSeq instrument (Illumina Inc. San Diego, CA, USA) for a paired-end 2 × 280 cycles run using a standard v3 cartridge according to the manufacturer’s instructions. As an internal quality run control, we used a spike-in of 15% PhiX. After de-multiplexing of the samples by the MiSeq Reporter software (Illumina Inc. San Diego, CA, USA) the analysis of the read sequences was performed by a Visual Basic-based in-house software approach (BloodGroup Analyser, Institute of Transplantation Diagnostics and Cell Therapeutics (ITZ), University Hospital of Düsseldorf, Düsseldorf, Germany) considering quality control values and high coverage to automate data analysis. Algorithms were developed to distinguish between sequencing artifacts such as cross-over products and closely related alleles. Allele differentiation was generally performed by comparison of exons 2 and 3 only. In the case of DQA1*03:02 and DQA1*03:03 allele distinction, the amino acid substitution occurs in the non-sequenced exon 1. Here, we used the intron 2 substitution c.331+4T>C as a surrogate marker (Supplementary Figure S1).

### 2.3. Calculations and Statistics

Odds ratios were estimated for comparison of haplotype frequencies between healthy controls and patients with type 1 diabetes. *p*-values and confidence intervals were calculated using Fisher´s exact tests and the Baptista–Pike method. *p*-values of two-sided tests ≤ 0.05 were considered statistically significant. GraphPad Prism 9 (Version 9.1.0, GraphPad Software) was used for statistical analyses.

## 3. Results

### 3.1. Identification of the HLA-DQA1*03:03 Allele in the Type 1 Diabetes DQ8 Risk Haplotype

In our study, 229 children and adolescents with type 1 diabetes were HLA genotyped at their *HLA-DRB1, -DQA1* and -*DQB1* loci in order to further characterize well-described HLA class II risk haplotypes. By extended sequencing of exon 3 in addition to exon 2, we identified variations in the *DQA1* alleles in addition to previously described HLA haplotypes associated with increased type 1 diabetes risk. In addition to the DQA1*03:01 allele, we detected the DQA1*03:03 allele within the DR4/DQ8 haplotypes (Table 1). For DRB1*04:01–DQB1*03:02, combinations with both DQA1*03:01 or DQA1*03:03 were detected (Table 1). In contrast, DRB1*04:05–DQB1*03:02 was solely combined with the newly described DQA1*03:03 allele (Table 1). Besides being part of the known type 1 diabetes-associated DR4/DQ8 risk haplotypes, DQA1*03:03 was also found in six other haplotypes which have not been associated with an altered type 1 diabetes risk so far (Supplementary Table S1). Overall, DQA1*03:03 was detected in 10.04% (*n* = 46) of all haplotypes. From these 46 DQA1*03:03 allele carriers, 30 comprised the putative DR4/DQ8 risk haplotypes. Detailed information about all haplotypes and their respective frequencies found in our cohort are given in Supplementary Table S1. To unravel a potential phenotypic variability deriving from homo- and heterozygosity of the HLA-DQA1*03:03 allele, we also assessed the HLA genotype of the individuals carrying this allele. However, all individuals carrying the DQA1*03:03 allele in the DR4/DQ8 risk haplotype (*n* = 30) were heterozygous at this particular MHC loci (data not shown Supplementary Table S2). These results indicated that, in contrast to previously described studies, two distinct *DQA1* alleles contributed to the DR4/DQ8 type 1 diabetes risk haplotype.

### 3.2. HLA-DQA1*03:03 Characteristics

DQA1*03:01 as well as DQA1*03:03 encodes the α chain of the DQ antigen, which forms a heterodimer together with the ß chain coded by *DQB1*. DQA1*03:01 and DQA1*03:03 belong to the same serologically defined group but differ in their protein sequence; specifically, HLA-DQA1*03:03 differs from DQA1*03:01 by a single nucleotide substitution in exon 3 at codon 160 (c.548C>A; Supplementary Figure S2). This substitution leads to a nonsynonymous amino acid change from Alanine (A) to Aspartic Acid (D) at amino acid position 160 when compared to DQA1*03:01 (Supplementary Figure S3) and has potential implications on the structure of the protein complex [9].

### 3.3. DR4 Haplotypes Containing HLA-DQA1*03:03

We next investigated the distribution of DQA1*03:01 and DQA1*03:03 in the DR4/DQ8 haplotypes of the general population. For this reason, we chose selected donors from the Bone Marrow Donor Registry Düsseldorf, Germany, with different DRB1*04-DQB1*03:02 haplotypes and screened them for the presence of DQA1*03:03 or DQA1*03:01. A total of 279 samples with 19 different DRB1*04 alleles were analyzed (Table 2). In accordance with the haplotypes found in type 1 diabetes patients, all DRB1*04:05 carriers had the DQA1*03:03 allele, whereas the DRB1*04:01 carriers were found to have either the DQA1*03:03 (14.3%) or the DQA1*03:01 allele (85.7%; Table 2). In addition, we identified three further DRB1*04 alleles (DRB1*04:07, DRB1*04:08 and DRB1*04:10) that were partly combined with DQA1*03:03 (Table 2). DRB1*04:10 only occurred together with DQA1*03:03, and the alleles DRB1*04:07 and DRB1*04:08 were combined with either DQA1*03:01 or DQA1*03:03 (Table 2). With these results, we were able to estimate the proportion of DQA1*03:03 in DR4/DQ8 haplotype carriers in the general population.

### 3.4. Comparison of HLA-DQA1*03:03 Containing Type 1 Diabetes Risk Haplotypes between Healthy Controls and Individuals with Type 1 Diabetes

To investigate the impact of the newly described haplotypes (including DQA1*03:03) on type 1 diabetes susceptibility, we next compared risk haplotype frequencies between the pediatric type 1 diabetes cohort (*n* = 229) and a control cohort of 9786 individuals. These healthy donors had been DRB1–DQB1 HLA-typed previously (haplotype frequencies in Supplementary Table S3). The distribution of DQA1*03:01/DQA1*03:03 alleles in DR4/DQ8 haplotypes of this control population was here estimated using the DQA1*03:03 proportions of sequenced DR4/DQ8 positive donors. According to these results, 100% of DRB1*04:05 carriers and 14.3% of the DRB1*04:01 carriers were estimated to carry the DQA1*03:03 allele, respectively (Table 2). Odds ratios (OR) were calculated for every haplotype. Here, we found that both DRB1*04:01–DQB1*03:02 haplotypes including either DQA1*03:01 or DQA1*03:03 were associated with increased type 1 diabetes susceptibility with similar ORs of 6.18 and 4.90, respectively (Table 3). The DRB1*04:05–DQB1*03:02 comprising DQA1*03:03 also had a significant association with susceptibility to type 1 diabetes (OR of 15.36). Furthermore, we were able to confirm the previously described type 1 diabetes risk association of the DR3/DQ2 haplotype (OR of 4.04) and of the DRB1*04:02 or DRB1*04:04/DQ8 haplotypes (OR of 5.09 and 1.83, respectively). These results underline the type 1 diabetes risk association of known risk haplotypes when associated with the newly reported DQA1*03:03 allele.

## 4. Discussion

In this study we report the occurrence of a previously undescribed *DQA1* allele within the DR4 DQ8 haplotype predisposing for type 1 diabetes. Due to the common practice of HLA class II genotyping being limited to exon 2, as recommended by the international Type 1 Diabetes Genetics Consortium [8], this allele had not been detected previously. Exon 2 *HLA-DQA1* and *HLA-DQB1* encode the extracellular α1 and β1 domains that form the peptide-binding groove exerting the antigen binding and presenting function [2]. Most of the polymorphic sites in these exons affect the antigen interaction sites built by the so-called ‘anchor residues’ of the antigen-binding pocket [10]. By extended *DQA1* sequencing including exon 3, encoding the α2 domain, we now report the *DQA1* allele DQA1*03:03 to be part of the type 1 diabetes high risk DR4/DQ8 haplotype. The exon 3-coded α2 and β2 domains form an extracellular heterodimer not directly involved in peptide binding. However, these domains are thought to play a role in the complementary and effector functions of MHC class II molecules [11]. In particular, involvement in the binding of CD4 co-receptor molecules and homodimerization of MHC class II αβ heterodimers have been discussed [11]. Homodimerization of two MHC class II molecules bound to the same antigenic peptide occurs upon ligation by T cell receptor molecules and facilitates the formation of immunological synapses [12,13]. For HLA-DQA1*03:03, it was suggested that the amino acid substitution from Alanine (A) to Aspartic Acid (D) stabilizes dimerization of α2/β2 heterodimers by introducing a salt bridge instead of a hydrogen bond [11]. Against this background, we compared type 1 diabetes susceptibility between HLA-DQA1*03:01 or HLA-DQA1*03:03 carriers, and our results revealed no major differences in type 1 diabetes risk. Both DQ8 combinations were significantly increased in type 1 diabetes patients with only minor differences in odds ratios. 

Whereas the majority of studies to date assign only the HLA-DQA1*03:01 allele to the DQ8 serotype, there exist already a few publications that deviate from this. Klitz et al. [14] as well as Zhao et al. [15] report the DQA1*03:02 allele (besides DQA1*03:01) to be combined with DQB1*03:02. DQA1*03:02 differs from DQA1*03:03 by one amino acid substitution in codon 5 encoded by exon 1. Although we did not sequence exon 1, we were able to distinguish both alleles by a surrogate marker in intron 2, thus clearly identifying DQA1*03:03. Klitz et al. did not investigate polymorphisms outside exon 2 and was, therefore, not capable of identifying the DQA1*03:03 allele [14]. In future studies, the presence of the DQA1*03:03 allele needs to be carefully monitored and its possible relevance to other autoimmune diseases should be assessed.

Taken together, our study identified the HLA-DQA1*03:03 allele in well-described HLA type 1 diabetes risk haplotypes and thus helps to correct current inconsistencies in type 1 diabetes risk haplotype nomenclature.

## Figures and Tables

**Table 1:** HLA class II high risk haplotypes formerly described and found in our study.

	Erlich et al., 2008 Noble et al., 2012 Noble et al., 2015			Enczmann et al., 2021		
**Haplotype**	**DRB1**	**DQA1**	**DQB1**	**DRB1**	**DQA1**	**DQB1**
DR3/DQ2	03:01	05:01	02:01	03:01	05:01	02:01
DR4/DQ8	04:01	03:01	03:02	04:01	03:01	03:02
				04:01	03:03	03:02
	04:02	03:01	03:02	04:02	03:01	03:02
	04:04	03:01	03:02	04:04	03:01	03:02
	04:05	03:01	03:02			
				04:05	03:03	03:02

**Table 2:** DQA1*03:03 haplotype counts in selected DR4/DQ8 haplotypes of the general population.

DRB1	DQA1	DQB1	Haplotype Counts	Proportion of DQA1*03:03 within DR04:x [%]
04:01	03:01	03:02	18	
04:01	03:03	03:02	3	14.3
04:02	03:01	03:02	38	0
04:03	03:01	03:02	26	0
04:04	03:01	03:02	38	0
04:05	03:03	03:02	22	100
04:06	03:01	03:02	29	0
04:07	03:01	03:02	27	
04:07	03:03	03:02	7	20.6
04:08	03:01	03:02	3	
04:08	03:03	03:02	8	72.7
04:10	03:03	03:02	1	100
04:11	03:01	03:02	7	0
04:13	03:01	03:02	17	0
04:14	03:01	03:02	16	0
04:15	03:01	03:02	7	0
04:21	03:01	03:02	2	0
04:26	03:01	03:02	3	0
04:28	03:01	03:02	2	0
04:36	03:01	03:02	1	0
04:38	03:01	03:02	1	0
04:50	03:01	03:02	3	0

**Table 3:** DRB1–DQA1–DQB1 haplotype counts and frequencies in controls and type 1 diabetes patients.

Haplotype	DRB1	DQA1	DQB1	Haplotype Counts (Frequencies, %) of Controls	Haplotype Counts (Frequencies, %) of T1D Patients	Odds Ratio (95% CI)	*p*-Value
DR3/DQ2	03:01	05:01	02:01	2049 (10.5)	147 (32.1)	4.04 (3.31–4.95)	<0.0001
DR4/DQ8	04:01	03:01	03:02	696 (3.6)	85 (18.6)	6.18 (4.85–9.94)	<0.0001
	04:01	03:03	03:02	116 (0.6)	13 (2.8)	4.90 (2.80–8.62)	<0.0001
	04:02	03:01	03:02	192 (1.0)	22 (4.8)	5.09 (3.27–7.97)	<0.0001
	04:04	03:01	03:02	429 (2.2)	18 (3.9)	1.83 (1.13–2.91)	0.023
	04:05	03:03	03:02	49 (0.3)	17 (3.7)	15.36 (8.62–26.52)	<0.0001

Estimated haplotype counts and frequencies among control subjects (*n* = 9786) and type 1 diabetes patients (*n* = 229), estimated odds ratios and *p* values are listed.

## Data Availability

The datasets generated during and/or analyzed during the current study are available from the corresponding author upon reasonable request.

## Supplementary Materials

The following are available online at https://www.mdpi.com/article/10.3390/genes12121879/s1, Figure S1: *HLA-DQA1* intron 2 sequence alignment, Figure S2: *HLA-DQA1* exon 2 and 3 sequence alignment, Figure S3: *HLA-DQA1* peptide sequence alignment, Table S1: DRB1 DQA1 DQB1 haplotype counts and frequencies in 229 type 1 diabetes patients, Table S2: Genotypes of T1D patients carrying the *DQA*03:03* allele in the DR4/DQ8 risk haplotype, Table S3: DRB1 DQB1 haplotype frequencies in 9786 controls.
