# Supplementary material for: Next Generation Sequencing Identifies the HLA-DQA1*03:03 Allele in the Type 1 Diabetes Risk-Associated HLA-DQ8 Serotype
Source: Genes (Basel). 2021 Nov 25;12(12):1879. doi: 10.3390/genes12121879 (PMC8701008; doi:10.3390/genes12121879)
Supplement: Supplementary file 1 [file genes-12-01879-s001.zip › genes-1431344-supplementary.pdf]

```
gDNA                        4110       4120       4130       4140       4150       4160       4170       4180       4190       4200
 DQA1*03:02:01:01            GTAC GTGTCCACCA TTCTGCCTTT CTTTACTGAT TTATCCCTTT ATACCAAGTT TCATTATTTT CTTTCCAAGA GGTCCCCAGA TCTTCTCATG
 DQA1*03:02:01:02            ---- ---------- ---------- ---------- ---------- ---------- ---------- ---------- ---------- ----------
 DQA1*03:03:01:01            ---T ---------- ---------- ---------- ---------- ---------- ---------- ---------- ---------- ----------
 DQA1*03:03:01:02            ---T ---------- ---------- ---------- ---------- ---------- ---------- ---------- ---------- ----------
 DQA1*03:03:01:03            ---T ---------- ---------- ---------- ---------- ---------- ---------- ---------- ---------- ----------
 DQA1*03:03:01:04            ---T ---------- ---------- ---------- ---------- ---------- ---------- ---------- ---------- ----------
 DQA1*03:03:01:05            ---T ---------- ---------- ---------- ---------- ---------- ---------- ---------- ---------- ----------
 DQA1*03:03:01:06            ---T ---------- ---------- ---------- ---------- ---------- ---------- ---------- ---------- ----------
 DQA1*03:03:01:07            ---T ---------- ---------- ---------- ---------- ---------- ---------- ---------- ---------- ----------
 DQA1*03:03:01:08            ---T ---------- ---------- ---------- ---------- ---------- ---------- ---------- ---------- ----------
 DQA1*03:03:01:09            ---T ---------- ---------- ---------- ---------- ---------- ---------- ---------- ---------- ----------
 DQA1*03:03:01:10            ---T ---------- ---------- ---------- ---------- ---------- ---------- ---------- ---------- ----------
 DQA1*03:03:01:11            ---T ---------- ---------- ---------- ---------- ---------- ---------- ---------- ---------- ----------
 DQA1*03:03:01:12            ---T ---------- ---------- ---------- ---------- ---------- ---------- ---------- ---------- ----------
 DQA1*03:03:01:13            ---T ---------- ---------- ---------- ---------- ---------- ---------- ---------- ---------- ----------
 DQA1*03:03:01:14            ---T ---------- ---------- ---------- ---------- ---------- ---------- ---------- ---------- ----------
 DQA1*03:03:01:15            ---T ---------- ---------- ---------- ---------- ---------- ---------- ---------- ---------- ----------
 DQA1*03:03:01:16Q           ---T ---------- ---------- ---------- ---------- ---------- ---------- ---------- ---------- ----------
 DQA1*03:03:03               ---T ---------- ---------- ---------- ---------- ---------- ---------- ---------- ---------- ----------
 DQA1*03:03:04               ---T ---------- ---------- ---------- ---------- ---------- ---------- ---------- ---------- ----------
 DQA1*03:03:05               ---T ---------- ---------- ---------- ---------- ---------- ---------- ---------- ---------- ----------
```

Supplementary Figure S1:
The HLA-DQA1 intron 2 sequence alignment identifies one point mutation distinguishing two HLA-DQA1*03:02 allelels from 19 HLA-DQA1*03:03 (c.331+4T>C). Sequence alignment was downloaded from (https://www.ebi.ac.uk/ipd/imgt/hla/).

```
DQA1*03:01:01:01    CT GAC CAT GTT GCC TCT TAC GGT GTA AAC TTG TAC CAG TCT TAT GGT CCC TCT GGG CAG TAC AGC CAT GAA TTT
DQA1*03:03:01:01    -- --- --- --- --- --- --- --- --- --- --- --- --- --- --- --- --- --- --- --- --- --- --- --- ---

AA Codon            30                  35                  40                  45                  50
DQA1*03:01:01:01   GAT GGA GAC GAG GAG TTC TAT GTG GAC CTG GAG AGG AAG GAG ACT GTC TGG CAG TTG CCT CTG TTC CGC AGA TTT
DQA1*03:03:01:01   --- --- --- --- --- --- --- --- --- --- --- --- --- --- --- --- --- --- --- --- --- --- --- --- ---

AA Codon            55                  60                  65                  70                  75
DQA1*03:01:01:01   AGA AGA TTT GAC CCG CAA TTT GCA CTG ACA AAC ATC GCT GTG CTA AAA CAT AAC TTG AAC ATC GTG ATT AAA CGC
DQA1*03:03:01:01   --- --- --- --- --- --- --- --- --- --- --- --- --- --- --- --- --- --- --- --- --- --- --- --- ---

AA Codon            80                  85                   90                  95                 100
DQA1*03:01:01:01   TCC AAC TCT ACC GCT GCT ACC AAT G|AG GTT CCT GAG GTC ACA GTG TTT TCC AAG TCT CCC GTG ACA CTG GGT CAG
DQA1*03:03:01:01   --- --- --- --- --- --- --- --- -|-- --- --- --- --- --- --- --- --- --- --- --- --- --- --- --- ---

AA Codon           105                 110                 115                 120                 125
DQA1*03:01:01:01   CCC AAC ACC CTC ATC TGT CTT GTG GAC AAC ATC TTT CCT CCT GTG GTC AAC ATC ACC TGG CTG AGC AAT GGG CAC
DQA1*03:03:01:01   --- --- --- --- --- --- --- --- --- --- --- --- --- --- --- --- --- --- --- --- --- --- --- --- ---

AA Codon           130                 135                 140                 145                 150
DQA1*03:01:01:01   TCA GTC ACA GAA GGT GTT TCT GAG ACC AGC TTC CTC TCC AAG AGT GAT CAT TCC TTC TTC AAG ATC AGT TAC CTC
DQA1*03:03:01:01   --- --- --- --- --- --- --- --- --- --- --- --- --- --- --- --- --- --- --- --- --- --- --- --- ---

AA Codon           155                 160                 165                 170                 175
DQA1*03:01:01:01   ACC TTC CTC CCT TCT GCT GAT GAG ATT TAT GAC TGC AAG GTG GAG CAC TGG GGC CTG GAT GAG CCT CTT CTG AAA
DQA1*03:03:01:01   --- --- --- --- --- -A- --- --- --- --- --- --- --- --- --- --- --- --- --- --- --- --- --- --- ---

AA Codon           180
DQA1*03:01:01:01   CAC TGG G
DQA1*03:03:01:01   --- --- -
```

Supplementary Figure S2:
The HLA-DQA1 exon 2 and 3 sequence alignment identifies one point mutation distinguishing HLA-DQA1*03:03:01:01 from HLA-DQA1*03:01:01:01. The point mutation is located in codon 160 (c.548C>A) in exon 3. Sequence alignment was downloaded from https://www.ebi.ac.uk/cgi-bin/ipd/imgt/hla/align.cgi, Release 3.45.0 (2021-07-12)

```
AA Pos.                  -21        -11         -1         10         20         30         40         50         60         70
 DQA1*03:01:01:01           MIL NKALMLGALA LTTVMSPCGG EDIVADHVAS YGVNLYQSYG PSGQYSHEFD GDEEFYVDLE RKETVWQLPL FRRFRRFDPQ FALTNIAVLK
 DQA1*03:03:01:01           --- ---------- ---------- ---------- ---------- ---------- ---------- ---------- ---------- ----------

 AA Pos.                    80         90        100        110        120        130        140        150        160        170
 DQA1*03:01:01:01    HNLNIVIKRS NSTAATNEVP EVTVFSKSPV TLGQPNTLIC LVDNIFPPVV NITWLSNGHS VTEGVSETSF LSKSDHSFFK ISYLTFLPSA DEIYDCKVEH
 DQA1*03:03:01:01    ---------- ---------- ---------- ---------- ---------- ---------- ---------- ---------- ---------D ----------

 AA Pos.                   180        190        200        210        220        230
 DQA1*03:01:01:01    WGLDEPLLKH WEPEIPTPMS ELTETVVCAL GLSVGLVGIV VGTVLIIRGL RSVGASRHQG PL
 DQA1*03:03:01:01    ---------- ---------- ---------- ---------- ---------- ---------- --
```

Supplementary Figure S3:
The HLA-DQA1 peptide sequence alignment identifies one mismatch distinguishing HLA-DQA1*03:03:01:01 from HLA-DQA1*03:01:01:01. The amino acid substitution is located in amino acid position 160 (A>D). Sequence alignment was downloaded from https://www.ebi.ac.uk/cgi-bin/ipd/imgt/hla/align.cgi, Release 3.45.0 (2021-07-12)

Supplementary Table S1. DRB1 DQA1 DQB1 haplotype counts and frequencies in 229 T1D patients

DRB1	DQA1	DQB1	Haplotype count	frequency (%)
01:01	01:01	05:01	32	7.0
01:01	01:02	05:04	1	0.2
01:02	01:01	05:01	6	1.3
03:01	05:01	02:01	147	32.1
04:01	03:01	03:02	85	18.6
04:01	03:03	03:01	6	1.3
04:01	03:03	03:02	13	2.8
04:02	03:01	03:02	22	4.8
04:03	03:01	03:05	1	0.2
04:04	03:01	03:02	18	3.9
04:05	03:03	02:02	4	0.9
04:05	03:03	03:02	17	3.7
04:08	03:02	03:01	1	0.2
04:08	03:03	03:01	1	0.2
04:08	03:03	03:04	1	0.2
04:28	03:01	03:02	1	0.2
07:01	02:01	02:02	15	3.3
07:01	02:01	03:03	1	0.2
07:03	02:01	02:02	1	0.2
07:05	03:03	02:02	1	0.2
08:01	04:01	04:02	11	2.4
08:01	04:04	04:02	1	0.2
08:04	04:01	04:02	1	0.2
09:01	03:02	03:03	3	0.7
09:01	03:03	02:02	3	0.7
11:01	05:05	03:01	7	1.5
11:02	05:01	02:01	1	0.2
11:03	05:05	03:01	2	0.4
11:04	05:05	03:01	3	0.7
12:01	05:05	03:01	3	0.7
13:01	01:03	06:03	8	1.7
13:02	01:02	06:04	20	4.4
13:02	01:02	06:09	2	0.4
14:04	01:04	05:03	2	0.4
15:01	01:02	05:02	1	0.2
15:01	01:02	06:02	2	0.4
15:01	01:03	05:03	1	0.2
15:02	01:03	06:01	1	0.2
16:01	01:02	05:02	9	2.0
16:02	01:02	05:02	2	0.4
07:KY	03:01	02:02	1	0.2

Supplementary Table S2. Genotypes of T1D patients carrying the *DQA*03:03* allele in the DR4/DQ8 risk haplotype (n=30)

Haplotype 1			Haplotype 2		
DRB1	DQA1	DQB1	DRB1	DQA1	DQB1
11:03	05:05	03:01	04:01	03:03	03:02
01:01	01:01	05:01	04:01	03:03	03:02
01:01	01:01	05:01	04:01	03:03	03:02
03:01	05:01	02:01	04:01	03:03	03:02
03:01	05:01	02:01	04:01	03:03	03:02
03:01	05:01	02:01	04:01	03:03	03:02
03:01	05:01	02:01	04:01	03:03	03:02
03:01	05:01	02:01	04:01	03:03	03:02
03:01	05:01	02:01	04:01	03:03	03:02
03:01	05:01	02:01	04:01	03:03	03:02
03:01	05:01	02:01	04:01	03:03	03:02
03:01	05:01	02:01	04:01	03:03	03:02
03:01	05:01	02:01	04:01	03:03	03:02
13:02	01:02	06:04	04:05	03:03	03:02
16:01	01:02	05:02	04:05	03:03	03:02
13:02	01:02	06:04	04:05	03:03	03:02
15:01	01:02	06:02	04:05	03:03	03:02
07:01	02:01	02:02	04:05	03:03	03:02
04:08	03:03	03:01	04:05	03:03	03:02
04:02	03:01	03:02	04:05	03:03	03:02
03:01	05:01	02:01	04:05	03:03	03:02
03:01	05:01	02:01	04:05	03:03	03:02
03:01	05:01	02:01	04:05	03:03	03:02
03:01	05:01	02:01	04:05	03:03	03:02
03:01	05:01	02:01	04:05	03:03	03:02
03:01	05:01	02:01	04:05	03:03	03:02
03:01	05:01	02:01	04:05	03:03	03:02
03:01	05:01	02:01	04:05	03:03	03:02
03:01	05:01	02:01	04:05	03:03	03:02
03:01	05:01	02:01	04:05	03:03	03:02

Supplementary Table S3. DRB1 DQB1 haplotype frequencies in 9786 controls

DRB1	DQB1	frequency (%)
01:01	05:01	9,560
01:01	05:02	0,005
01:01	05:03	0,015
01:01	05:04	0,138
01:01	06:02	0,010
01:01	06:04	0,005
01:02	05:01	1,349
01:02	05:03	0,005
01:03	03:01	0,077
01:03	05:01	0,256
01:07	05:01	0,005
03:01	02:01	10,469
03:01	03:01	0,036
03:01	03:02	0,015
03:01	03:03	0,010
03:01	03:19	0,010
03:01	04:02	0,005
03:01	06:02	0,020
03:02	02:01	0,005
03:02	02:03	0,005
03:02	04:02	0,015
03:04	02:01	0,015
03:07	02:01	0,005
03:08	02:02	0,005
04:01	02:02	0,007
04:01	03:01	2,617
04:01	03:02	4,151
04:01	04:02	0,005
04:02	03:01	0,005
04:02	03:02	0,981
04:02	05:01	0,005
04:03	02:01	0,005
04:03	03:01	0,026
04:03	03:02	0,674
04:03	03:04	0,072
04:03	03:05	0,133
04:03	04:02	0,010
04:04	02:02	0,005
04:04	03:02	2,192
04:04	04:02	0,113
04:05	02:01	0,005

04:05	02:02	0,082
04:05	03:01	0,020
04:05	03:02	0,250
04:05	04:01	0,026
04:05	04:02	0,015
04:06	03:02	0,005
04:06	04:02	0,046
04:07	03:01	0,755
04:07	03:02	0,021
04:07	03:04	0,005
04:08	03:01	0,276
04:08	03:02	0,010
04:08	03:04	0,138
04:08	03:19	0,005
04:10	04:02	0,010
04:11	03:02	0,005
04:13	03:02	0,005
04:14	03:02	0,005
04:15	03:02	0,005
04:21	03:02	0,005
07:01	02:01	0,056
07:01	02:02	8,921
07:01	03:01	0,046
07:01	03:02	0,005
07:01	03:03	3,500
08:01	03:01	0,028
08:01	03:02	0,050
08:01	04:02	2,529
08:01	04:04	0,005
08:01	06:02	0,005
08:02	03:01	0,005
08:02	03:02	0,015
08:02	04:02	0,092
08:03	03:01	0,343
08:03	03:02	0,005
08:03	06:01	0,031
08:03	06:04	0,005
08:04	03:01	0,051
08:04	03:19	0,005
08:04	04:02	0,179
08:06	03:01	0,005
08:06	06:02	0,010
08:11	04:02	0,005
08:12	04:02	0,005

09:01	02:02	0,010
09:01	03:02	0,005
09:01	03:03	0,776
09:01	03:04	0,005
10:01	05:01	0,838
10:01	06:02	0,005
11:01	02:01	0,010
11:01	03:01	8,087
11:01	03:02	0,011
11:01	05:02	0,026
11:01	06:02	0,005
11:01	06:03	0,005
11:01	06:04	0,005
11:02	03:01	0,118
11:02	03:19	0,138
11:02	06:03	0,005
11:03	03:01	0,981
11:03	06:02	0,005
11:03	06:04	0,005
11:04	03:01	3,581
11:04	03:02	0,005
11:04	04:02	0,005
11:04	06:02	0,005
11:04	06:03	0,010
11:09	03:01	0,005
11:11	03:01	0,010
11:12	03:01	0,026
11:13	05:03	0,020
11:14	03:01	0,020
11:15	03:01	0,026
11:19	03:01	0,005
11:27	03:01	0,010
12:01	02:02	0,003
12:01	03:01	1,888
12:01	03:03	0,005
12:02	03:01	0,061
12:02	06:01	0,005
13:01	03:01	0,026
13:01	05:01	0,016
13:01	05:03	0,010
13:01	06:01	0,005
13:01	06:02	0,036
13:01	06:03	7,188
13:02	03:02	0,005

13:02	05:01	0,087
13:02	06:02	0,010
13:02	06:03	0,010
13:02	06:04	3,725
13:02	06:09	0,479
13:02	06:41	0,005
13:02	06:84	0,005
13:03	02:02	0,005
13:03	03:01	1,390
13:04	03:19	0,005
13:05	03:01	0,072
13:14	03:01	0,015
13:16	06:04	0,005
13:18	06:03	0,005
13:19	03:01	0,005
13:22	06:02	0,005
13:28	06:03	0,005
14:01	05:01	0,010
14:01	05:02	0,010
14:01	05:03	0,501
14:02	03:01	0,010
14:03	03:01	0,005
14:04	05:03	0,168
14:06	03:01	0,015
14:07	05:03	0,036
14:11	05:03	0,005
14:12	03:01	0,010
14:16	05:03	0,010
14:54	05:01	0,010
14:54	05:02	0,031
14:54	05:03	2,080
14:54	06:01	0,005
14:54	06:02	0,020
15:01	02:01	0,005
15:01	03:01	0,010
15:01	05:01	0,051
15:01	05:02	0,235
15:01	05:03	0,015
15:01	06:01	0,082
15:01	06:02	12,073
15:01	06:03	0,251
15:01	06:04	0,005
15:01	06:13	0,005
15:02	05:01	0,015

15:02	05:02	0,005
15:02	05:03	0,015
15:02	06:01	1,017
15:02	06:02	0,005
15:03	06:02	0,005
15:03	06:03	0,010
15:04	06:02	0,005
15:06	05:02	0,010
16:01	03:02	0,005
16:01	05:01	0,005
16:01	05:02	2,509
16:01	06:03	0,010
16:02	03:01	0,010
16:02	05:02	0,220
